# Management of salivary gland adenoid cystic carcinoma: institutional experience of a case series

**DOI:** 10.1590/S1516-31802006000100006

**Published:** 2006-01-05

**Authors:** Alfio José Tincani, André Del Negro, Priscila Pereira Costa Araújo, Hugo Kenzo Akashi, Antonio Santos Martins, Albina Milani Altemani, Gilson Barreto

**Keywords:** Head and neck cancer, Salivary gland neoplasms, Adenoid cystic carcinoma, Neoplasm staging, Radiotherapy, Carcinoma adenóide cístico, Estadiamento de neoplasias, Radioterapia, Neoplasias de cabeça e pescoço, Neoplasias das glândulas salivares

## Abstract

**CONTEXT AND OBJECTIVE:**

Salivary gland tumor management requires long-term follow-up because of tumor indolence and possible late recurrence and distant metastasis. Adenoid cystic carcinoma (ACC) accounts for 10-15% of such tumors. The aim here was to evaluate surgical and clinical management, staging and follow-up of ACC patients in one academic institution.

**DESIGN AND SETTING:**

Retrospective study at Head and Neck Service, Universidade Estadual de Campinas.

**METHODS:**

Dataon 21 patients treated between 1993 and 2003 were reviewed. Management utilized clinical staging, histology and imaging. Major salivary gland tumor extent was routinely assessed by preoperative ultrasonography. Diagnosis, surgery type, margin type (negative/posi- tive), postoperative radiotherapy and recurrence (presence/absence) were evaluated.

**RESULTS:**

There were eleven major salivary gland tumors (52.3%), seven submandibular and four parotid. Ten patients (47.7%) had minor salivary gland ACC (all in palate), while the submandibular was the most frequently affected major one. Diagnoses were mostly via fine-needle aspiration (FNA) and incision biopsy. Frozen sections were used for six patients. There was good ultrasound/FNA correlation. Sixteen (76%) had postoperative radiotherapy. One (4.7%) died from ACC and five now have recurrent disease: three (14.2%) locoregional and two (9.5%) distant metastases.

**CONCLUSION:**

Adenoid cystic carcinoma has locally aggressive behavior. In 21 cases, of ACC, the facial nerve was preserved in all except in the few with gross tumor involvement. Treatment was defined from physical examination, imaging, staging and histology.

## RESUMO

**CONTEXTO E OBJETIVO:**

Tumores de glândulas salivares exigem acompanhamento clínico por longos períodos devido à evolução indolente dessas neoplasias e à possibilidade tardia de recidivas locais e metástases a distância. O carcinoma adenóide cístico (CAC) compreende 10% a 15% dos tumores de glândulas salivares. O objetivo deste trabalho é analisar a conduta terapêutica, o estadiamento e o acompanhamento de pacientes com CAC em cabeça e pescoço em instituição de ensino.

**TIPO DE ESTUDO E LOCAL:**

Estudo retrospectivo realizado no Serviço de Cirurgia de Cabeça e Pescoço, Universidade Estadual de Campinas.

**MÉTODOS:**

Revemos o tratamento do CAC de 21 pacientes tratados entre 1993 e 2003. Os pacientes foram estadiados conforme os critérios clínicos da União Internacional Contra o Câncer (UICC) de 1998 e o tratamento foi baseado na avaliação clínica e imagenológica.

**RESULTADOS:**

11 tumores estavam localizados nas glândulas salivares maiores (52,3%), sendo sete em glândula submandibular e quatro em parótida. Em 10 pacientes (47,7%), os tumores estavam em glândulas salivares menores (todos em palato). Quando ocorreram em glândula salivar maior, a submandibular foi a mais acometida. O diagnóstico foi realizado quase sempre por punção aspirativa por agulha fina (PAAF) ou biópsia. Biópsia por congelação no intraoperatório foi realizada em seis pacientes. Obtivemos boa correlação entre o ultra-som e a PAAF. Em 16 pacientes (76%), a radioterapia pós-operatória foi realizada. Um dos pacientes (4,7%) foi a óbito pelo CAC e cinco tiveram recidivas da doença, sendo três locoregionais (14,2%) e dois (9,5%) por metástases a distância.

**CONCLUSÃO:**

Carcinoma adenóide cístico apresenta comportamento agressivo localizado. Em 21 casos, o nervo facial foi preservado, exceto nos raros pacientes com invasão grosseira pelo tumor. O tratamento baseou-se no exame físico, exames de imagem, estágio do tumor e diferenciação histológica.

## INTRODUCTION

Salivary gland neoplasms form a diverse group of tumors, with different histological characteristics and clinical behavior patterns.^1-12^ This marked variation in histological grading and clinical classification means that the evaluation of these tumors requires extensive knowledge of anatomy and physiology, as well as expertise in pathology. Long-term followup of patients with salivary gland neoplasms is mandatory^1-9,11^ because of the frequently indolent but relentlessly infiltrative behavior associated with late locoregional recurrence and distant metastasis.

Approximately 10-1 5% of salivary gland tumors are adenoid cystic carcinomas (ACC).^1,2-9^ This type of neoplasm was first described by Billroth in 1856 as a benign neoplasm and was named cylindroma for its cribriform appearance formed by tumor cells with cylindrical pseudolumina or pseudospaces. ACC occurs most often in minor salivary glands and the submandibular gland, and less frequently in the sublingual and parotid glands.^4,11^ Other rare locations include the aerodigestive tract, minor salivary glands, lachrymal glands and adnexal skin glands. Most ACC patients are in their fifth and sixth decades of life, and females are slightly more affected than males.^1,4,11^

**Figure 1:**
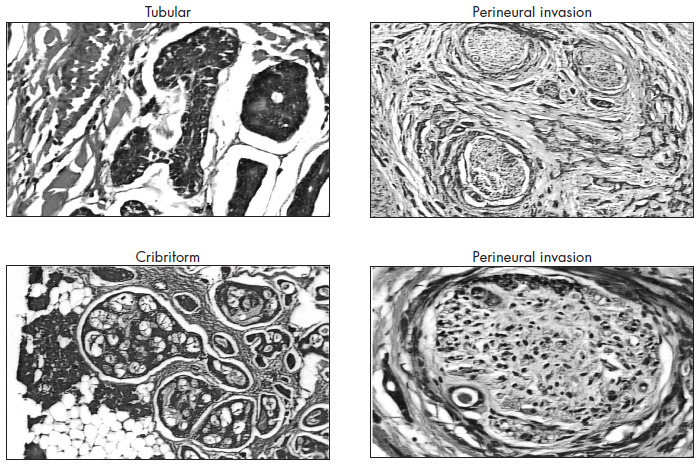
Histology of adenoid cystic carcinoma subtypes (tubular and cribriform) and perineural invasion (400 x magnification and hematoxylin-eosin staining).

Three histological subtypes of ACC are known: cribriform, tubular and solid. They may occur either separately or together in the same tumor, and the solid subtype is the most aggressive^1,4,12^ (Figure 1). Perineural invasion is characteristic of these tumors^1-12^ and occurs in up to 60% of cases.^6^ Cervical metastasis is rare and occurs in only 8-13% of patients.^3,7,11^ Distant metastasis may occur in up to 50% of ACC patients during the course of the disease, with the lungs and bones as the most common sites.^3,5^

This paper reviews the management of ACC in the Head and Neck Service of the Department of Surgery in the teaching hospital of Universidade Estadual de Campinas (Unicamp), in order to evaluate its degree of aggressiveness in our experience.

## MATERIAL AND METHODS

From August 1993 to February 2003, 21 patients with ACC in the salivary glands were treated in the Head and Neck Service of the Department of Surgery at Universidade Estadual de Campinas (Unicamp). All patients whose treatment began before the start of this period were excluded from the present study.

Patients were classified according to their clinical stage, based on the 1998 criteria of the Union Internationale Contre le Cancer (UICC).

Imaging examinations were used in all cases. For early-stage major salivary gland tumors, ultrasound was the only imaging examination done. For minor salivary glands, all patients had preoperative computed tomography (CT) scans to determine the extent of the local tumor in the palate. For major salivary glands, four patients also needed a CT scan to allow better assessment of the lesions.

Diagnoses were made on the basis of histological samples, always preoperatively, by means of either fine-needle aspiration (in case of major salivary glands) or incision biopsy (minor salivary glands). Thus, frozen sections were only utilized to confirm the diagnosis or verify that the surgical margins were negative.

All of the patients underwent surgery with curative intent and sixteen (76%) received adjuvant radiotherapy, five after local recurrence. Surgical management was applied only after physical examination, imaging scans (mainly for minor salivary gland tumors), clinical staging and histological assessment of the tumor. The type of surgery was planned on the basis of the primary tumor site and the locoregional occurrence of metastasis, and it involved partial or total maxillectomy, parotidectomy and transoral resections. Patients with cervical metastasis underwent neck dissection according to the level of the metastasis, thus ranging from radical to selective neck dissection.

Positive or narrow margins, perineural invasion or more aggressive histological subtypes (generally the solid type) were the criteria for deciding to utilize postoperative radiotherapy, and this was employed in sixteen cases.

## RESULTS

The majority of the patients were female. For major salivary gland tumors, six patients (28.5%) were in stage I, three (14.3%) were in stage II, and two (9.4%) were in stage III. None of the patients had stage IV disease. For minor salivary gland tumors, seven patients (33.3%) were in stage I and three (14.3%) were in stage II. Two patients with submandibular ACC were N1.

The imaging scans accurately depicted the extent of the primary lesion: none of the scans underassessed the stage of the tumoral lesions, as confirmed by intraoperative findings. In four patients who had either T2 or T3 lesions, or tumors invading adjacent structures, a tomography scan was used to complement the preoperative assessment. For minor salivary gland tumors, this examination was done on all patients to allow better assessment of the degree of maxillary sinus invasion.

The diagnosing of major salivary gland tumors by aspirate biopsy was possible in 11 patients (52.3%) and in the case of one minor salivary gland tumor (4.7%). An incision biopsy was done in the cases of nine (42.8%) minor salivary gland tumors, and six cases (28.5%) required a frozen section biopsy during surgery. Table 1 describes the diagnosis and treatment of all 21 patients.

**Table 1:** Adenoid cystic carcinoma diagnosis treatment of 21 patients based on clinical stage: a case series report

Age; sex	Primary site	Diagnostic method	Surgery date; staging	Type of surgery	Subtype	Margin	Recurrence	Postoperative treatment	Follow-up
41 Female	Submandibular gland	FNA	Jun 1993 T2N0M0	Wide resection	Tubular	Wide	None	Rtx	FOD
60 Female	Hard palate	Incision biopsy	Aug 1993 T2N0M0	Maxillectomy	Cribriform	Narrow	None	Rtx	FOD
48 Male	Hard palate	FNA	Mar 1994 T1N0M0	Transoral resection	Tubular	Wide	None	None	FOD
51 Female	Hard palate	Incision biopsy	May 1995 T3N0M0	Wide resection	Solid	Narrow	Sep 1998; Oct 2000	Maxillectomy + Rtx	Active disease
60 Female	Hard palate	Incision biopsy	Aug 1995 T2N0M0	Maxillectomy	Cribriform	Narrow	Jan 1999	Rtx	FOD
32 Female	Submandibular gland	FNA	Dec 1995 T2N1M0	Wide resection + SOND	Cribriform	Wide	None	Rtx	FOD
64 Male	Parotid gland	FNA	Mar 1996 T2N0M0	Superficial parotidectomy	Solid	Wide	Aug 1997	Total parotidectomy + Rtx	FOD
46 Female	Parotid gland	Incision biopsy	Jun 1996 T2N0M0	Total parotidectomy	Tubular	Wide	None	Rtx	FOD
54 Male	Hard palate	Incision biopsy	Aug 1996 T2N0M0	Wide resection	Tubular	Wide	None	Rtx	FOD
26 Female	Submandibular gland	FNA/ Incision biopsy	Dec 1996 T2N1M0	Wide resection + SOND	Cribriform	Wide	None	Rtx	FOD
51 Female	Hard palate	Incision biopsy	May 1997 T3N0M0	Wide resection	Solid	Narrow	Sep 1998; Oct 2000	Maxillectomy + Rtx	Active disease
47 Female	Submandibular gland	FNA	Jun 1997 T3N0M0	Wide resection + SOND	Cribriform	Narrow	Nov 1998	Rtx; Ctx/Rtx	Death due to metastasis; Oct 2000
66 Male	Soft palate	Incision biopsy	Aug 1997 T2N0M0	Wide resection	Tubular	Wide	None	None	FOD
43 Female	Submandibular gland	FNA	Jun 1998 T3N0M0	Wide resection + SOND	Cribriform	Narrow	Feb 1999	Rtx; Ctx	Pulmonary metastasis
68 Male	Hard palate	Incision biopsy	Mar 1999 T3N0M0	Partial maxillectomy	Solid	Wide	None	Rtx + surgery	FOD
67 Female	Submandibular gland	FNA	Jan 2002 T2N0M0	Wide resection + SOND	Cribriform	Wide	None	Rtx	FOD
41 Female	Submandibular gland	FNA	Aug 2002 T1N0M0	Wide resection	Tubular	Narrow	None	None	FOD
60 Male	Parotid gland	FNA	Aug 2002 T1N0M0	Superficial parotidectomy	Tubular	Wide	None	None	FOD
71 Female	Parotid gland	Incision biopsy	Aug 2002 T3N0M0	Total parotidectomy	Solid	Wide	Mar 2003	Rtx + craniofacial	Active Disease/ Pulmonary Metastasis
58 Female	Hard palate	Incision biopsy	Oct 2002 T1N0M0	FNA	Cribriform	Wide	Jun 2003	Maxillectomy + Rtx	FOD
72 Male	Hard palate	Incision biopsy	Feb 2003 T1N0M0	Wide resection	Tubular	Wide	None	None	FOD

*FNA = fine-needle aspiration; FOD = free of disease; Rtx = radiotherapy; Ctx = chemotherapy; SOND = Supraomohyoid neck dissection.*

Nine patients had tumors that showed perineural invasion, which is a common trend in this type of neoplasm^6^ that confirms its locally aggressive behavior. Tumors were located in the major salivary glands in 11 patients (52.3%), of which four were in the parotid and seven in the submandibular glands. Ten patients (47.7%) had minor salivary gland tumors, all in the palate, one of them in the soft palate.

Among the parotid tumors, three were located in the superficial lobe and one in the deep lobe. Facial nerve resection was necessary in three patients, one in the main trunk and two in the mandibular and cervical branches, all due to local invasion.

Among the submandibular tumors, one case showed hypoglossal nerve invasion and resection was done for oncological purposes. Four cases required neck dissections (two selective and two radical).

All but one patient had a confirmed preoperative diagnosis of malignant salivary gland tumor. One patient had a preoperative aspirate biopsy that showed pleomorphic adenoma, but the final pathology report confirmed ACC. Among the frozen section biopsies for six patients with malignant lesions, ACC was accurately diagnosed in four patients (66.6%). The pathology reports revealed six close surgical margins (less than 2 mm): two in submandibular tumors and four in minor salivary gland tumors.

The pathology reports identified the tubular subtype of ACC in eight cases (38%), the cribriform subtype in eight (38%) and the solid subtype in five (24%).

Six patients had local recurrence and were surgically salvaged. Four had minor salivary gland tumors of the palate and were treated with radical^1^ or partial^3^ maxillectomy. One patient had parotid ACC that required cranial-facial resection because of invasion of the skull base, probably as a result of trigeminal nerve invasion.

One patient (4.7%), who had a T3N0 submandibular tumor, died of the disease 40 months after surgical treatment because of distant metastasis. Five patients had recurrence: three (14.2%) present with locoregional disease, one in the skull base and another in the maxillary sinus recurrence. Two patients (9.5 %) now present distant pulmonary metastasis.

## DISCUSSION

Salivary gland ACC has indolent behavior, but is prone to late local recurrence.^1,8,11,12^ The management of the 21 patients in this series was based on the clinical stage, histological subtype and ancillary imaging examinations. There were no T4 primary tumors among our patients. This finding, together with the relatively short follow-up in this series, meant that there were few distant metastases, in contrast to other reports.^3-5,7-9^ There was a slight female predominance among the cases (66.6%), which was similar to what has been described in the literature from around the world.^1,4,11^ Most ACC occurred in minor salivary glands and, when present in the major salivary glands, the submandibular gland was the one most frequently affected. There were no cases with ACC in the sublingual glands.

**Figure 2:**
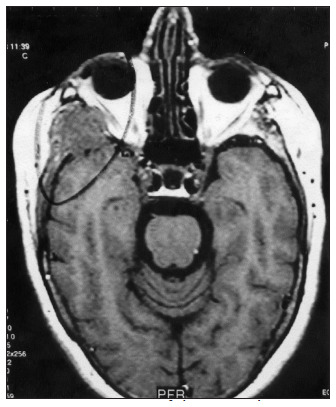
Invasion of the central nervous system by adenoid cystic carcinoma as shown in magnetic resonance imaging.

According to the literature, the primary treatment of ACC is surgery with wide mar- gins.^1,2,4,9^ In the present series, for parotid tumors, superficial parotidectomy was the standard procedure for stage I disease. Total parotidectomy was done when there was clinical invasion or suspicions of local tumor features. Preservation of the facial nerve was attempted in all cases, but the main trunk was sacrificed in one case because of gross tumor invasion (T3 lesion) that later showed invasion of the skull base and brain parenchyma (Figure 2). In two T2 lesions, the cervicofacial trunk was also sacrificed because of tumor invasion.

Although nerve grafting is currently indicated when the facial nerve is resected,^1,8,9^ we only used grafting in cases of T1 and T2 tumors, in patients ≤ 60 years old, or when radiotherapy was not planned for the immediate postoperative period. No grafting was done in patients with a sacrificed nerve because of these patients’ advanced age and clinical stage.

Wide margins were always possible for submandibular gland tumors, while for T1 and T2 lesions the adjacent level II lymph nodes were resected *en bloc* during submandibulectomy. In cases of T3 lesions, submandibulectomy with selective neck dissection (including one case of supraomohyoid dissection) was done because the level II lymph nodes suffered from local tumor invasion, as confirmed by frozen section biopsies in N0 patients. In N+ patients (with level I and II lymph nodes), modified radical neck dissection was done, with the accessory nerve being spared. Selective neck dissection for salivary gland tumors is controversial in N0 patients and is of little benefit to these patients.^13,14^

The imaging examinations for major salivary gland tumors included ultrasound for all patients and CT scans only when invasion of the adjacent structures was suspected. Magnetic resonance imaging (MRI) was done in only one patient with invasion of the skull base and clearly showed neural invasion and its ascending route to the brain parenchyma (Figure 2).

For minor salivary gland ACC, all tumors were in the palate, with ten in the hard palate and one in the soft palate. Transoral resection was done on all T1 and T2 lesions with free margins up to 1 cm wide in order not to compromise the oncological safety of the surgery. Although the surgical field was limited in some cases, no close margins were reported. For larger tumors, maxillectomy was done by means of the Webber-Ferguson incision. No orbital exanteration was needed.

CT scans were done for all minor salivary gland ACC in order to determine the tumor stage more precisely, to plan proper surgical intervention, and as a reference for future comparisons. Surgical margins were analyzed and, when close or positive, radiotherapy was indicated. Adjuvant radiotherapy was used for all cases of T3 tumors, even when the margins were free of disease. The portals were wide and encompassed all nerve routes because of the tendency of the tumors to show perineural invasion, as seen in nine of the 21 patients (42.8%).

## CONCLUSIONS

Adenoid cystic carcinoma is an indolent tumor with locally aggressive behavior and a high rate of local recurrence, especially when perineural invasion is present. Radiotherapy is mandatory when disease-free margins cannot be obtained surgically and when there is locally advanced disease or high-grade histological findings. Long-term follow-up is needed because local recurrence and distant metastasis may occur late in the course of disease, especially among high-risk patients.
